# Correlated Diffusion of Colloidal Particles near a Liquid-Liquid Interface

**DOI:** 10.1371/journal.pone.0085173

**Published:** 2014-01-20

**Authors:** Wei Zhang, Song Chen, Na Li, Jia zheng Zhang, Wei Chen

**Affiliations:** 1 State Key Laboratory of Surface Physics, Department of Physicse, Fudan University, Shanghai, China; 2 Department of Applied Physics, Northwestern Polytechnical University, Xi'an, China; 3 Department of Physics, Jinan University, Guangzhou, China; 4 Kavli Institute for Theoretical Physics China, CAS, Beijing, China; University of California, Irvine, United States of America

## Abstract

Optical microscopy and multi-particle tracking are used to investigate the cross-correlated diffusion of quasi two-dimensional colloidal particles near an oil-water interface. The behaviors of the correlated diffusion along longitudinal and transverse direction are asymmetric. It is shown that the characteristic length for longitudinal and transverse correlated diffusion are particle diameter 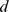 and the distance 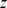 from particle center to the interface, respectively, for large particle separation 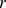. The longitudinal and transverse correlated diffusion coefficient 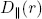 and 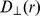 are independent of the colloidal area fraction 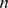 when 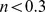, which indicates that the hydrodynamic interactions(HIs) among the particles are dominated by HIs through the surrounding fluid for small 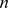. For high area fraction 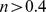, the power law exponent for the spatial decay of 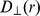 begins to decrease, which suggests the HIs are more contributed from the 2D particle monolayer self for large 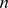.

## Introduction

The dynamic behavior of confined colloidal suspensions has recently received a considerable amount of attentions [1]–[6]. Microfluidic devices, porous media, fluid interface or cell membrane [7]–[15] are real-world circumstances in which particles are usually spatially confined. Colloid behavior in spatially confined environments is more complicated than that in unbounded three-dimensional (3D) fluid bulk. The correlated diffusions coefficient 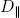 and 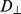 between a pair of particles in 3D bulk are well known as 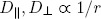 and 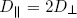 [16]. The hydrodynamic interactions (HIs) between a pair of particles confined by solid walls decay with the inter-particle separation 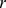 as 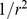 [4], [17], [18]. The behaviors of the longitudinal and transverse correlated diffusion of particles confined by a two-dimensional (2D) interface or viscous membrane are different [2], [16]–[21]: 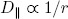 and 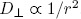, which is caused by the break in the spatial symmetry.

The diffusion behaviors of particles reflect the influence of the boundary conditions [22]–[26]. Solid wall condition with a non-slip boundary [27], which could cut off the fluid field in its vicinity, has been studied extensively. However, there are comparatively few experimental studies on the influences of the fluid-fluid interface, which could partially transforms the surrounding flow field, on the correlated diffusion of particles. Ref. [28] investigated the influence of a fluid-fluid interface on correlated diffusion and presented a scaling method to yield a master curve capturing a transition from 3D bulk to the interface dominated. While the colloidal monolayer in 3D bulk approaches to the interface, the dynamic responses of the correlated diffusion along the two normal direction are asymmetric, which need to be further investigated in detail.

In this paper, we report an experimental measurement of the cross-correlated diffusion of colloidal particles near a water-decahydronaphthalene (decalin) interface. It is shown that for larger particle separation, along the line connecting the centers of the particles and the direction perpendicular to this line, the characteristic length is particle diameter 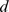 and the distance between particle monolayer and the interface 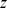, respectively. The influence of a fluid-fluid interface on the dynamic behavior of the colloidal monolayer tends to saturate, when the particle separation is much larger than the distance between the interface and the monolayer. These phenomena reveal that the hydrodynamics interactions (HIs) of monolayer particles occur through the colloidal monolayer self and the surrounding fluid, and the weights of HIs through these different paths (the colloidal monolayer or its surrounding fluid) change with the particle separation. In addition, we measured the correlated diffusion of particles for high colloidal density. In the case of colloidal area fraction 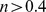, 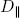 and 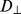 begin to depend on 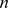, which suggests HIs through colloidal monolayer dominate even for large particles separation.

## Materials and Methods

Two types of colloidal particles, both with a diameter 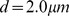, were used: polystyrene (PS) latex spheres and silica spheres. The silica and PS spheres were purchased from Bangs and Invitrogen, respectively. PS microspheres with sulfate functional groups on the surface are negatively charged. The surface functional groups of silica sphere is Si-OH. The PS and silica samples were suspended in deionized water of 18.2 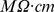. By centrifugation, we cleaned the samples seven times to eliminate any possible surfactant before use.

**Figure 1:**
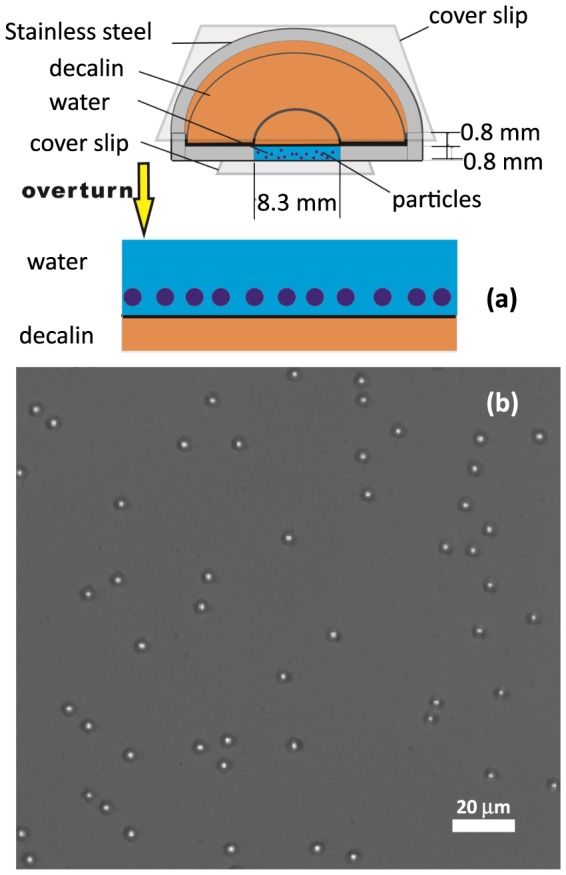
(color online) (a) A schematic of the process of the sample preparation. (b) An optical microscope image of the silica spheres (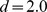 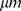) suspended near the water-decalin interface at an area fraction of 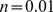 (note that the water is on the top and the decalin is on the bottom).

The experimental setup is shown as in Fig. 1a. The sample cell is composed of a stainless steel disk with a hole of the diameter 8.3 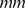, and the bottom of this hole is sealed with a 0.1-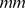-thick glass cover slip, that also serves as an optical window. In addition, the inner container of the cell is divided into two layers, as shown in Fig. 1a. The cleaned water-sphere suspension was filled into the bottom layer, and then, decalin (a mixture of cis and trans with a density of 0.896 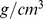) purchased from Sigma-Aldrich was added to the top of the suspension, completely filling the inner container. We cover the top of the inner container with another cover slip. We let the cell settle upside-down for two hours after flipping quickly. The fluids in the cell are completely pinned by the surrounding solid boundaries. Thus, the water layer could remain on the top and the oil will stay on the bottom. With the aid of gravity, colloidal particles fall onto the water-decalin interface and form a monolayer, as shown in Fig. 1a. All DI water was used within 30 min after preparation. The colloidal particles preferentially remain in the water rather than sink into decalin for they are hydrophilic. Particles remain in the medium (water) with the higher dielectric constant and the image charge in decalin has same sign as the surface charge of the particles [29]. Due to Coulomb repulsion, particles keep a certain distance away from the interface. The influence from cover slips can be neglected because the distance between the cover slip and the colloidal monolayer is about 0.8 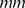 and sufficiently large.

Using an inverted Olympus IX71 microscope, the motion of the colloidal particles was recorded by a digital camera (Prosilica GE1050) at a rate of 14 frames per second. Each image sequence comprises 500 consecutive frames (taken over 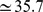 sec) and each image is 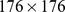 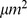. From these image sequences, we obtain the particle positions and construct their trajectories with a homemade software. The center positions of particle were calculated using the geometric average of pixels with gray-degree weight. Generally, the positions could be also obtained using 2D surface peak fitting with a higher spatial resolution than using the geometric average of pixels, up to within 0.1 pixel. Our calculation shows the results obtained using two methods agree within 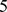 per cent deviation. This is because the particles are highly symmetrical and include enough pixels (more than 100 pixels per particle). The real length of a pixel is approximately 170 nm. The spatial resolution was estimated to be third or half pixel, approximately 60–100 nm.

## Results and Discussion

### Positions of the particle monolayers

**Figure 2:**
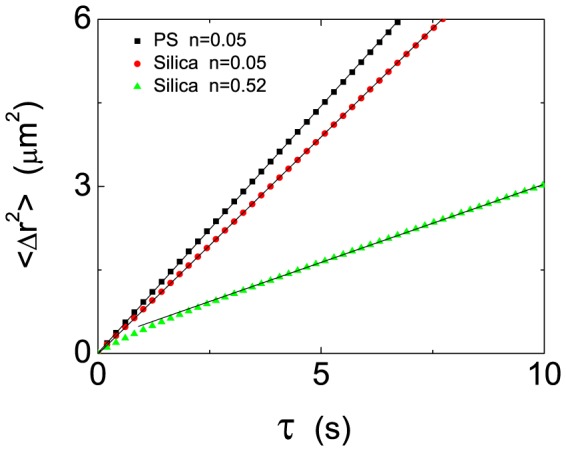
(color online) The curves of single-particle mean square displacement (MSD) for PS spheres and silica spheres. The area fractions 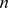 are 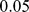 (square black symbols) for PS spheres, 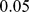 (dot red symbols)and 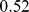 (triangle green symbols) for silica spheres.

**Figure 3:**
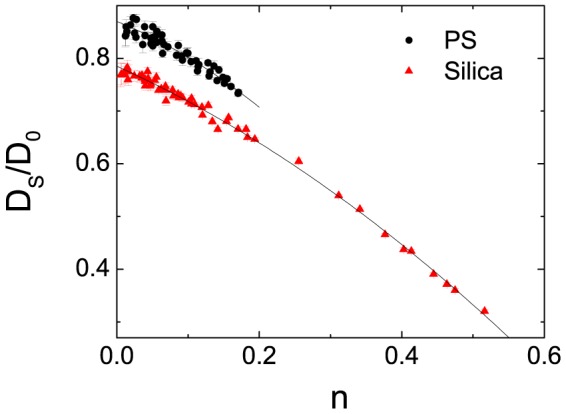
(color online) The measured self-diffusion coefficient 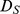 scaled by 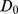 as a function of the particle area fraction 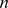 for silica and PS spheres. Different symbols represent data for different particles. The solid lines show the second-order polynomial fitting 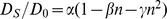.

The PS monolayer is located at a higher position above the oil-water interface than the silica monolayer because the PS spheres are less dense and have a greater surface charge than the silica particles. The average distance 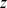 between the particle monolayer and the interface can be estimated by measuring the single diffusion coefficient of the particles. The single-particle mean square displacement (MSD) 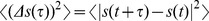 was calculated from the particle trajectories 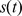. The MSD curves with typical different 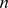 are plotted in Fig. 2. According to the equation 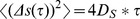, we obtained the long time self-diffusion coefficient 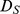 for different particle area fractions 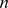. Figure 3 exhibits the relationship of the diffusion coefficient 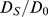 to 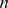 for the PS and silica spheres, where 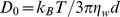 is the diffusion coefficient of a single particle in water. We fit the results with the second-order polynomial,
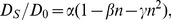
(1)which is shown in Fig. 3 as the solid lines. Where 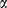, 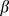 and 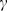 are the fitting parameters, whose fitted values are given in Table I.

**Table 1:** The distance 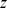 from particle's center to the interface and the fitted values of the 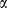, 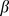 and 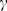 in Fig. 3. Here 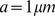 is the radius of the particles.

Sample	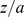	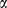	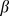	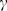
PS	2.3 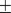 0.2	0.87 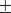 0.01	0.62 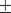 0.12	1.54 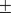 0.61
Silica	1.4 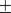 0.1	0.77 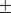 0.01	0.78 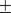 0.02	0.78 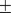 0.03

The parameter 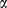 represents the local viscosity experienced by a single sphere at the dilute limit, whose value can be used to estimate the distance between the interface and the particle monolayer 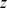. The distance from a particle's center to the interface 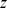 can be written as [26], [30]:
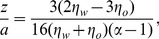
(2)where 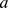 is the particle radius, 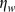 and 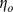 are the viscosity of water and decalin, respectively. Substituting the fitted values of 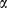 into Eq. 2, we obtain the distance 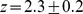 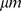 for PS spheres and 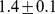 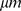 for silica spheres.

### Cross-correlated diffusion coefficient 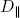 and 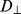


**Figure 4:**
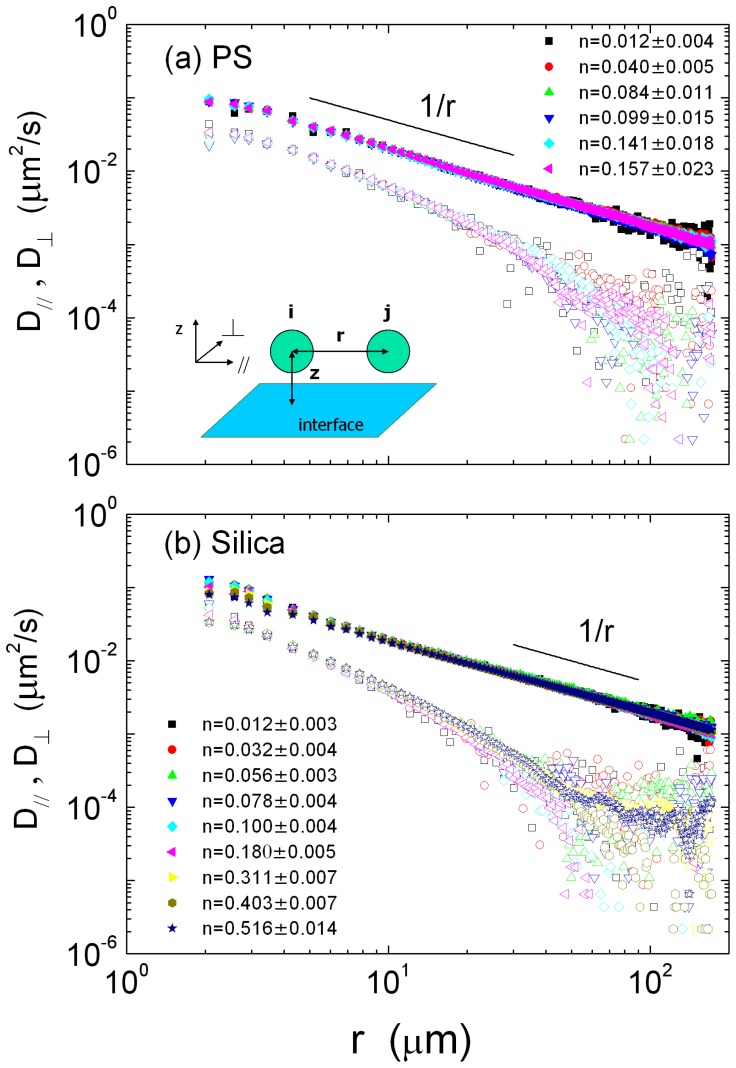
(color online) The measured correlated diffusion coefficient 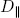 (solid symbols) and 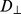 (open symbols) as a function of the inter-particle distance 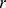 with various values of the area fraction 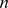 for (a) PS and (b) silica spheres. The solid lines with slopes 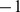 are visual guides. The geometry of measuring the correlated diffusion is depicted in the inset.

Tracking individual particle's trajectory, we obtain particle's cross-correlated motion via the ensemble of averaged tensor products of the particle's displacements [16]:
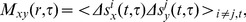
(3)where 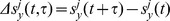. The superscript 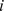 and 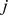 indicate distinct particle, and the subscript 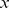 and 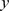 represent different coordinates, and 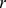 is the separation between particles 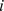 and 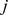 (as shown in the inset of Fig. 4). We focus only on the diagonal elements of the tensor product, 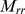 and 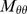 for the off-diagonal elements are uncorrelated. The diagonal element 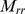 indicates the correlated motion along the line connecting the centers of the particles (longitudinal), and 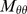 represents the correlated motion perpendicular to this line (transverse). It is shown that for a small time lag 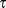, the measured correlated motions 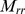 and 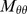 are linear functions of 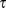. And, the cross-correlated diffusion coefficients are defined as 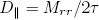 and 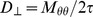.

Typically,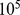 particle pairs were used to calculate the diffusion coefficients 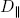 and 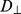 in Fig. 3. For example, with the area fraction (n = 0.01), 10 short movies were acquired and each movies containing 500 frames. 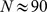 particles were found in each frame. The number of particle pairs used for the averaged diffusion curve is 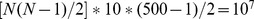. Because each diffusion curve usually contains 100 data points, each data point is thus averaged over 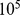 particle pairs. These numbers vary slightly among the colloidal samples with different concentrations. As Savin and Doyle[31] suggested, averaging of 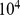 data points is enough to reduce the statics error in most cases. Because the exposition time to capture a frame was 30 ms, and the diffusivity of particles during that period is approximately 50 nm. The dynamics error could be also safely neglected.

Figure 4 exhibits 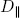 and 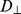 of the PS and silica spheres as functions of 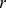 for different area fraction 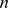. Each curve of a fixed 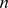 was obtained by averaging at least 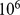 particle positions. The deviation of each 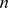 was given in the legend of Fig. 4. The behaviors of 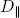 and 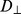 of the PS and silica monolayer are qualitatively similar. With the increase of 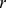, 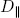 and 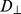 of the PS and silica spheres decrease in the form of power law 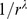. In the case of area fraction 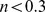, the curves of 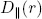 or 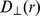 for different 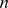 almost lie on a single curve respectively, i.e., the particle area fraction has little effect on their cross-correlated motion. While 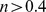, the power law exponent (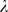, called the decay rate in the later part of the paper) of 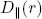 and 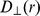 decrease with the increase of 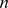, as shown in Fig. 4(b).

The mechanism behind the independence of 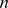 mentioned above is in line with the theoretical description given by Oppenheimer 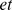 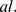 in Ref. [21]. In the particle monolayer, the far-field response of the correlated particle motion mainly arises from the momentum diffusion through the 3D surrounding fluid. The viscosity of 3D fluid suspension is usually a function of volume fraction 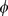 of particles. In our system 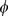 can be regarded as almost zero, regardless any change in the area fraction 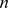 of the monolayer. Thus, the viscosity of the 3D fluid almost keeps as a nearly constant. The far-field 3D HIs between the particles are independent of the area fraction 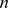, and as a result, the change of area fraction 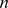 does not affect the cross-correlated diffusion 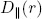 and 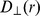. Indeed, Cui 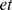 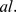 [17] also observed similar concentration independent effects for particles confined between two plates, but the reasons for their phenomena are different. In a confined particle monolayer, the fluid momentum is absorbed by the solid boundaries, and hence, the far-field fluid response arises solely from the mass-dipole perturbations, which are not influenced by the presence of neighboring particles.

### Influence of the interface

**Figure 5:**
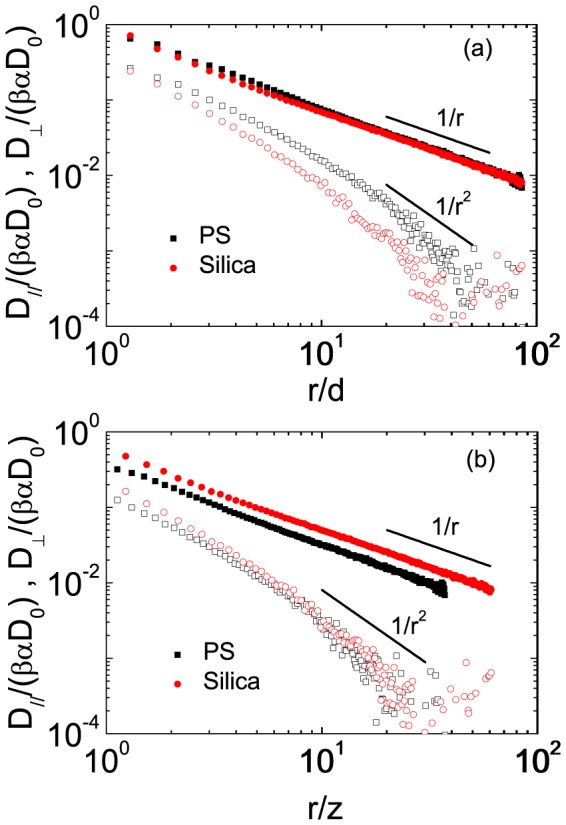
(Color online) (a) The mean correlated diffusion coefficient 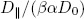 (solid symbols) and 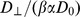 (open symbols) as a function of 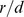 for PS and silica particles. (b) The mean correlated diffusion coefficient 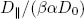 (solid symbols) and 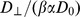 (open symbols) as a function of 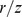 for PS and silica particles. The square represents data for the PS particles and the dots represent the silica particles. The solid lines with slopes 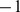 and 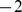 are visual guides.

To focus on the influence of the oil-water interface on the correlated diffusion coefficients, first, we averaged the curves of 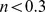 shown in Fig.4. Then, the averaged curves 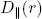 and 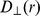 are normalized by the single diffusion constant 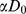. We found that the amplitude of 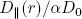 of the silica spheres is almost 1.2 times larger than that of the PS spheres, which number is just equal to the ratio of 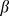 (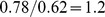, as displayed in Table I). This result is convincing because the parameter 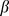 reflects the strength of the two-body HIs between two spheres, which shows that the results agree well for the measurements obtained by one or two particle diffusion methods. After the particle separation 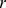 is normalized by particle's diameter 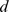, 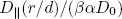 in different colloidal monolayer overlaps for large 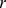, shown as in Fig. 5(a).

In the perpendicular direction, 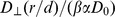 for PS and silica spheres deviates seriously. However, if we normalize the particle separation 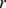 with the distance 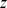, 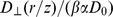 of different colloidal monolayers overlap for large separation (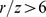), as shown in Fig. 5(b). Different methods to normalize the particle separation 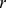 indicate that the characteristic length of longitudinal and transverse correlated diffusion is 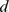 and 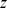, respectively, for far-field response.

We compare our results with previous studies for the following boundary conditions: colloidal spheres dispersed in an unbounded 3D bulk, confined by a solid wall or at an air-water interface [17]–[20]. We find that the cross correlated motion of colloidal spheres near an oil-water interface is similar to that at a viscous membrane [19], [20]: 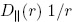 and 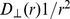 when 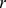 is large enough. For a viscous membrane, 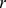 called “large enough” corresponds to 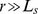; note that 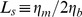 is the Saffman length [32], 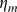 is the 2D membrane viscosity and 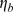 is the 3D bulk viscosity of the surrounding fluid. For our system, 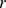 called “large enough” corresponds to 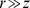 or 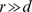 in different directions.

At first glance, the result that the decay rate of 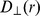 for the silica spheres is larger than that for the PS spheres seems to conflict with the fact that a larger decay rate should correspond to a smaller monolayer viscosity. Because the silica monolayer are closer to the interface, the silica spheres are located in a more viscous environment in comparison to the PS spheres. Thus, the decay rate of 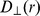 for the silica spheres should be less than that of the PS spheres. In fact, the viscosity 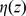 experienced by a sphere is the 3D viscosity of the local surrounding fluid, not the 2D viscosity 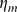 of the membrane. The influence of this surrounding viscosity 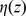 has been removed by 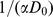 scaling, where 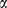 represents the effect of the local viscosity 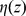. The finding that the value of the decay rate of 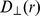 for the silica is larger than that of the PS spheres stems from the HIs that are modified by the interface. Because oil is more viscous than water, the flow field induced by the particles in water is suppressed by the oil-water interface. In addition, the HIs between particles at a distance 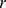 from each other decay rapidly when the monolayer is closer to the interface, which leads to a larger measured decay rate of 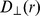 for a smaller 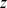.

### HIs through three paths

Similar to the mechanism of a membrane near a solid wall [19], the HIs among the particles in our system are transmitted through three paths: I) the 2D flow through the monolayer, II) the flow through the fluid layer sandwiched between the monolayer and the interface, III) the flows through the upper water layer and the below oil layer. Whereas the HIs through path I are dependent on 2D monolayer' viscosity 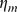, the HIs through paths II and III are dependent on the 3D viscosities 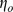 and 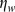 of the surrounding fluid.

The HIs through path I entail that the cross-correlated motion is a function of 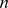; indeed, 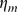 is a function of 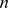 usually. The HIs through path I only contribute to the correlated motion when the separation 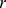 is less than the order of Saffman length 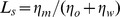 [32]. In our system, the viscosity 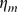 of the monolayer is very small, and we do not measured the 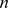 effects for 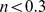. As 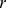 increases, the weight of the HIs through path I quickly decreases and the HIs through path II contribute to the correlated motion, rendering the correlated motion a function of 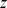 (Fig. 5). For a large separation 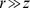, the HIs through path III are dominant. Because 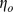 and 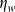 shows little change with the area fraction 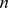, the correlated motion should be independent of 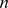 (Fig. 4 for 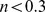). In addition, the decay rate of the correlated motion of the particles is independent of 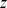 for 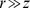. In the intermediate range of 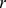, the relative weights of the HIs through paths II and III change gradually with 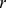. The decay rate of the cross-correlated motion shows a crossover tendency.

For a very high area fraction 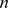, the averaged inter-particle separation permits an strong interaction between the particles, which means that the 2D monolayer viscosity 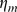 would become so large that the order of 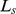 would be extended to the entire measuring range of 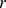 [28]. Then, the cross-correlated motion should be a function of 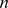, which may be supported by Fig. 4b: as the curves of 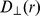 for 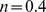 or 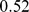 do shift upwards in comparison to those for 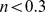.

## Conclusion

We experimentally investigated the cross-correlated diffusion of colloidal particles near an oil-water interface that distinctly affects the correlated diffusion coefficient 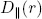 and 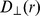. It is shown that the characteristic length of the longitudinal and transverse cross-correlated diffusion is particle diameter 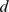 and the distance between the interface and the monolayer 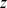, respectively, for far-field response. With the increase of particle separation 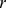, the influence of the interface on the cross-correlated diffusion changes, which indicates that the relative weights of the HIs through the colloidal monolayer self or through the surrounding fluid change gradually. The cross-correlated diffusions are independent of the colloidal area fraction 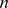 when 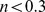, which indicates that the HIs among the particles are dominated by that through the surrounding fluid. For high area fraction 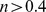, the decay rate of the cross-correlated diffusion begins to decrease with 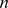, which suggests the HIs are more contributed from the 2D particle monolayer self.
